# Nitrogen and potassium limit fine root growth in a humid Afrotropical forest

**DOI:** 10.1038/s41598-024-63684-7

**Published:** 2024-06-07

**Authors:** Raphael Manu, Edzo Veldkamp, David Eryenyu, Marife D. Corre, Oliver van Straaten

**Affiliations:** 1 Department of Soil Science of Tropical and Subtropical Ecosystems, University of Göttingen, Göttingen, Germany; 2 Budongo Conservation Field Station, Masindi, Uganda; 3 Department of Green Chemistry and Technology, Ghent University, Ghent, Belgium; 4 Royal Zoological Society of Scotland (Edinburgh Zoo), Edinburgh, Scotland; 5 Northwest German Forest Research Institute, Göttingen, Germany

**Keywords:** Biogeochemistry, Budongo Forest, Lixisols, Nutrient limitations, Nutrient manipulation experiment, Uganda, Ecosystem ecology, Carbon cycle, Forest ecology

## Abstract

Nutrient limitations play a key regulatory role in plant growth, thereby affecting ecosystem productivity and carbon uptake. Experimental observations identifying the most limiting nutrients are lacking, particularly in Afrotropical forests. We conducted an ecosystem-scale, full factorial nitrogen (N)-phosphorus (P)-potassium (K) addition experiment consisting 32 40 × 40 m plots (eight treatments × four replicates) in Uganda to investigate which (if any) nutrient limits fine root growth. After two years of observations, added N rapidly decreased fine root biomass by up to 36% in the first and second years of the experiment. Added K decreased fine root biomass by 27% and fine root production by 30% in the second year. These rapid reductions in fine root growth highlight a scaled-back carbon investment in the costly maintenance of large fine root network as N and K limitations become alleviated. No fine root growth response to P addition was observed. Fine root turnover rate was not significantly affected by nutrient additions but tended to be higher in N added than non-N added treatments. These results suggest that N and K availability may restrict the ecosystem’s capacity for CO_2_ assimilation, with implications for ecosystem productivity and resilience to climate change.

## Introduction

Tropical forests play a critical role in the Earth’s biogeochemical cycles through their exchanges of carbon (C), water, and nutrients within the terrestrial biosphere. These forests store nearly 55% (471 ± 93 Pg C) of the world’s forest C pool compared to the 32% (272 ± 23 Pg C) in boreal and 14% (119 ± 6 Pg C) in temperate forests^1^; nearly one-third of the world’s soil C^2^ and 30–50% of terrestrial productivity^3^. Under increasing anthropogenic CO_2_ emissions, these forests are expected to heighten their potential for C sequestration to mitigate global climate change^4,5^. However, whereas higher atmospheric CO_2_ concentrations can improve plant growth, ecosystem productivity may be constrained by nutrient availability^6^. Most tropical forests are underlain by highly weathered soils^7^ and are expected to be deficient in essential rock-derived nutrients such as phosphorus (P)^8^, potassium (K)^9,10^, and calcium (Ca)^11^, as well as micronutrients^12^, that are required for primary productivity^13,14^. Nutrient limitations therefore assumes an important regulatory role in plant growth^15,16^, therein affecting ecosystem C capture and productivity. Despite reports of declining tropical forest C sink strengths^15,17–19^, how soil nutrients regulate CO_2_ assimilation across the vast tropical forest biome remains poorly understood^20^, particularly for understudied Afrotropical forests. Uncertainties remain whether or not terrestrial nutrient supply limit C sequestration now or in the future as global change effects intensify. Thus the current most limiting nutrient(s) to productivity in Afrotropical forests represents a significant biogeographical knowledge gap, and can affect these forests’ responses to herbivory^21^, disease infestation, and drought events^16,22–24^. Understanding the factors or nutrients that limit tree growth in these highly productive forests is necessary to predict changes in terrestrial carbon stocks and possible future threats to these ecosystems.

The productivity of most neotropical lowland forests growing on highly weathered soils, are reported to be pervasively P-limited^8,25,26^, particularly in central and eastern Amazonia^8^. The processes driving this P-limitation include: First, the generally low availability of P in tropical soils, triggered by the fixing of soil P to iron (Fe) and aluminium (Al) oxides and hydroxides, consequently rendering P occluded and less accessible for plant uptake. Second, the potentially rapid loss of rock-derived nutrients through leaching than can be replenished during further weathering, thereby constraining plant growth. To meet nutrient demand in such nutrient-impoverished soils^27^, plants may rely on symbiotic mycorrhizal associations or nutrient retention through efficient resorption of most limiting nutrients from senescing leaves. In contrasts to observations in the neotropics^8,25,26^, P limitation of productivity in Afrotropical forest remains unclear^9,16,28,29^, highlighting the biogeochemical heterogeneity of tropical forests^30^. On the other hand, a higher bioavailability of N than plant demand is commonly hypothesized in most tropical forests^7,31^. This claim was indirectly supported by the high abundance and diversity of N-fixing organisms in the tropics^7^; rapid soil N cycling rates^32^; high gaseous N losses^33^; high nitrate leaching^34^, and high foliar and litter N:P ratios^35^. In contrast to P and N, the role of K in ecosystem productivity has largely been overlooked in tropical forests and because of its monovalent charge, K^+^ ions can mostly be susceptible to leaching losses and become less available for plant use^36^.

Soils across the vast extent of tropical forests are heterogenous in pedogenesis^14^ mostly driven by parent material, topography, and climatic conditions, which in turn affects species composition, soil fertility, and the nutrient that potentially limit their productivity^13,27,37^. The rigorous evaluation of the latter has been made possible in recent decades through nutrient addition experiments^13^ but have been largely unrepresented in Afrotropical forests^38^. Whereas most tropical lowland fertilization experiments were situated on Oxisols and Ultisols, montane experiments were mostly located on Andisols, Inceptisols, and Histosols^38^, all of which were clayey in texture. There is a serious underrepresentation of experiments on sandy soils^38^ and in other regions with soils likely impoverished in rock-derived nutrients (such as Lixisols) due to strong weathering^38^. Lixisols are polygenetic soils which experienced strong weathering during earlier stages of pedogenesis under wetter past climates, then followed by depositions of base-rich aeolian dusts and ash from biomass burning^39^ and are commonly found in a transition zone between humid tropical forests and savannahs^9^.

Fine roots (≤ 2 mm diameter), which are responsible for water and nutrient acquisition, but are generally short-lived and non-woody, represent a functionally important part of belowground plant biomass^40^. Despite their critical functional roles (i.e., water and nutrient acquisition) in tree growth and ecosystem productivity, the overall mass contribution of fine roots to total net primary production (NPP) is relatively small (Table 1). Nonetheless, the production, decomposition, and turnover of fine roots remain an important pathway of soil organic C input and nutrient cycling^41^. The production of fine roots implies increased C allocation to roots, which can represent the balance between building new roots and maintaining metabolically older roots^42^, both of which require large resource investment by the trees. To this end, nutrient availability can exert major controls on fine-root dynamics^43^. The cost-benefit^44^ and optimal resource allocation^45^ theories suggest that plants will allocate additional biomass to organs that are resource-limited. Therefore, when soil nutrients are scarce, plants are likely to increase root lifespan (i.e., decrease turnover rate) to avoid nutrient loss^46^, and maintain a large network of fine root biomass (FRB)^45,47^ or decrease C allocation to fine roots as scarce nutrient(s) become adequately available^48,49^. Thus, fine-root responses to increased nutrient availability can serve as a diagnostic indicator of ecosystem nutrient limitation status^42,50^. This was evident in the decreased FRB and increased turnover in response to K fertilization^42^; decreased FRB with nitrogen (N) additions^50^ or with NPK additions^10^. Conversely, increased root productivity or root diameter were associated with P additions^8,51^ in central Amazonian forests growing on relatively P-impoverished soils^8,52^ particularly at total P < 300 mg P kg^−1^ where roots continue to exploratively extend to find P in the soil^53–55^. In a Panamanian lower-montane forest, FRB also increased with N additions^50^. Such increases in root biomass or productivity can be expected when prevailing nutrient limitations are not alleviated by experimental nutrient inputs or when the increased availability of a scarce nutrient induces the limitation of another^50,56^. These contrasting responses to nutrient addition also suggest links of fine root dynamics to site-specific drivers including soil fertility and species composition^57^. It is unclear how fine root growth in Afrotropical forest, on sandy soils, are likely to respond to elevated nutrient input in the face of increasing global change.

**Table 1:** Fine root contribution to net primary productivity (mean ± SE; Mg C ha^−1^ year^−1^) and responses of fine root biomass (FRB) or fine root production (FRP) to nutrient additions in tropical forests.

Location	Soil group (FAO classification)	NPP _total_	NPP_fine litter_	NPP_wood_	NPP_fine root_ (Soil depth)	Response to nutrient additions	References
Uganda	Lixisols	9.5 ± 0.9	6.4 ± 0.4	2.2 ± 0.4	1.2 ± 0.1 (30 cm)	FRB decreased with N and K; FRP decreased with K	This study; Manu et al.^9^
Panama	Cambisols/Nitisols	9.3	5.7	2.1	1.5 (20 cm)	FRB decreased with K	Wright et al.^10^; Yavitt et al.^42^; Sayer et al.^63^
Amazonia	Ferralsols	7.4 ± 0.2	4.4 ± 0.1	1.9	1.2 ± 0.1 (30 cm)	FRP increased with P	Cunha et al.^8^
Costa Rica	Cambisols	8.5	3.5	4.2 ± 1.1	0.8 ± 0.1 (15 cm)	FRP increased with P	Waring et al.^73^
Ecuador	Cambisols	–	1.8	–	3.2 (20 cm)	FRB decreased with P	Homeier et al.^85^
Ecuador	Acrisols	–	–	–	– (15 cm)	FRB increased with N, P, and K	Graefe et al.^86^
Panama	Andosols	8.3	4.4	2.3	1.6 (20 cm)	FRB increased with N	Adamek et al.^50^
Costa Rica	–	–	–	–	0.4 ± 0.0 (10 cm)	FRB-unresponsive to N and P	Alvarez et al.^64^

**Figure 1:**
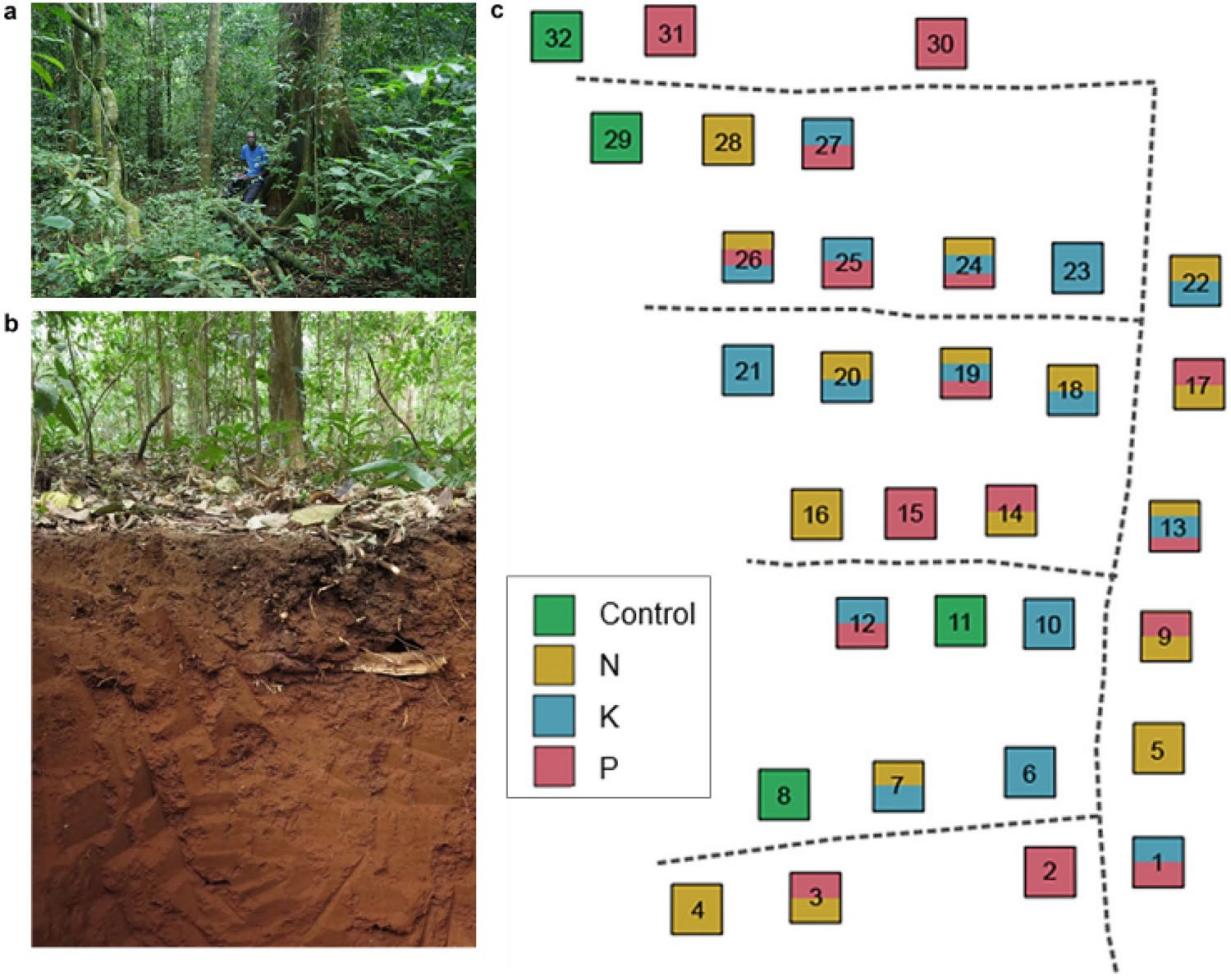
Vegetation (**a**), Lixisol soil (**b**), and layout of the full factorial N-P-K experiment (**c**) in the Budongo Forest Reserve, Uganda. The 32 experimental plots consist of eight randomly assigned treatments: Control, N, P, K, NP, NK, PK, and NPK with four replicates each. Nitrogen (125 kg N ha^−1^ year^−1^) was added as urea ((NH_2_)_2_CO), P (50 kg P ha^−1^ year^−1^) as triple superphosphate (Ca(H_2_PO_4_)_2_), and K (50 kg K ha^−1^ year^−1^) as muriate of potash (KCl) similar to an earlier experiment in Panama^10^. Plots are 40 m × 40 m in size and are at least 40 m apart, accessed using a walking trail (black dashed-line).

In this present study, we established an ecosystem-scale nutrient manipulation experiment (NME) on Lixisols in a humid semi-deciduous tropical forest in Uganda using a replicated full factorial experiment design (Fig. 1) to investigate which and whether nutrients (N, P, and K) limit fine root growth, and how rapid trees are able to respond to nutrient amendments in this Afrotropical ecosystem. We hypothesized that N and K will limit fine root growth, therefore, their increased availability will decrease FRB or FRP and increase fine root turnover rate. This prediction is based on earlier results from this site where both N and K were found to stimulate stem growth^9^, indicative of their potential limitations to plant function. Although our site has highly weathered substrate, the soil pH is near-neutral (Table 2) and suggestive that soil P will not be fixed by Fe and Al hydroxides, thus, P limitation of root growth will be unlikely at this site. We therefore predict that FRB and FRP will be irresponsive to P addition in this ecosystem. This experiment represents the first in a natural African tropical forest to experimentally evaluate N, P, and K limitation on fine root growth.

**Table 2:** Soil physical and biochemical characteristics (mean ± SE; n = 32 plots), measured in the Budongo Forest Reserve, Uganda, prior to the start of the nutrient addition experiment.

Site characteristics	Unit	Soil depth	
		0 − 10 cm	10 − 30 cm
Bulk density	g cm^−3^	1.2 ± 0.0	1.5 ± 0.0
Sand	%	55 ± 2	55 ± 2
Silt	%	27 ± 2	21 ± 1
Clay	%	18 ± 0.8	24 ± 1
Soil pH (1:2.5 H_2_O)		6.4 ± 0.0	6.1 ± 0.1
Soil organic carbon	kg C m^−2^	4.0 ± 0.1	4.6 ± 0.2
Total nitrogen	kg N m^−2^	0.4 ± 0.0	0.6 ± 0.0
C:N ratio		9.5 ± 0.1	8.0 ± 0.1
Total phosphorus	mg P kg^−1^	660 ± 104	506 ± 124
Calcium	mg Ca kg^−1^	6254 ± 362	5205 ± 180
Potassium	mg K kg^−1^	120 ± 6	52 ± 7
Magnesium	mg Mg kg^−1^	1410 ± 87	787 ± 83
Base saturation	%	98.2 ± 0.2	97.5 ± 1.0
Effective cation exchange capacity	mmol^+^ kg^−1^	149.2 ± 8.3	63.0 ± 4.1

## Results

### Responses of fine-root biomass and production to nutrient addition

**Figure 2:**
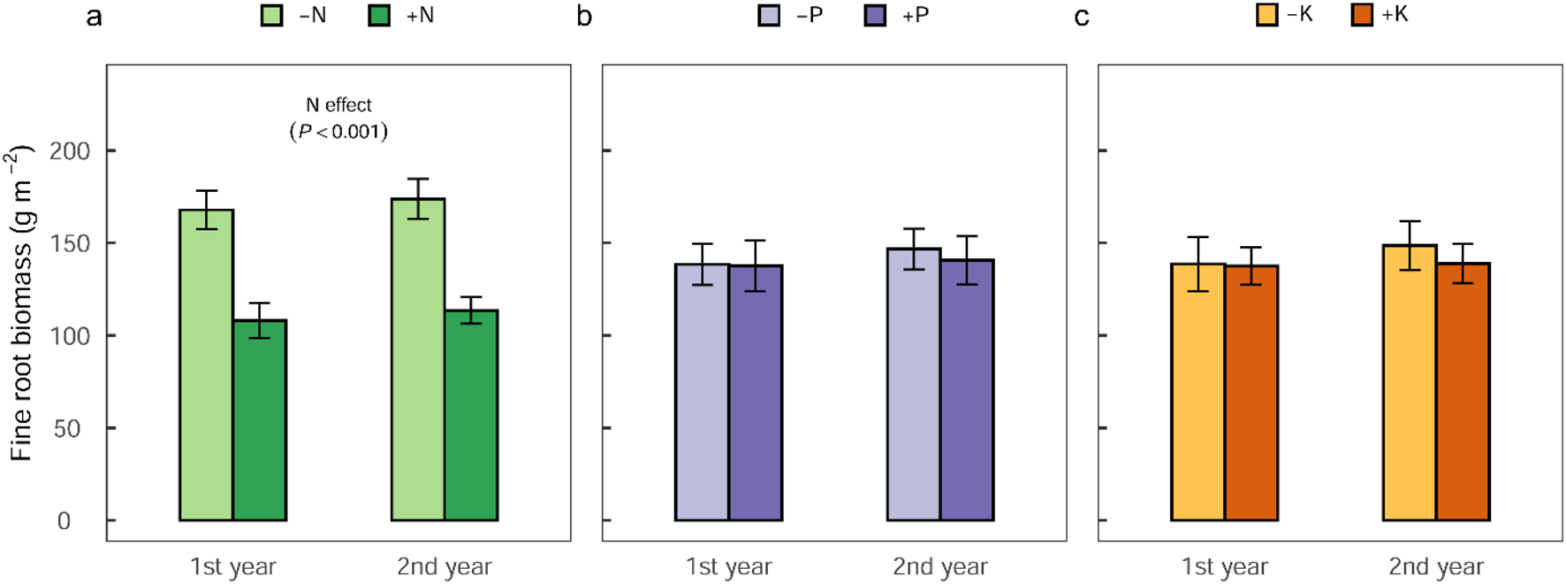
Nutrient addition effect on fine root biomass (mean ± SE, n = 16 plots), measured from six randomly located soil monoliths (20 cm × 20 cm × 10 cm) per plot after the first year (2019) and second year (2020) of the full factorial N-P-K experiment in the Budongo Forest Reserve, Uganda. Fine root biomass responses in (**a**) − N (Control, P, K, PK) compared to + N (N, NP, NK, NPK) treatments, (**b**) − P (Control, N, K, NK) compared to + P (P, NP, PK, NPK) treatments, and (**c**) − K (Control, N, P, NP) compared to + K (K, NK, PK, NPK) treatments. Fine root biomass decreased with N addition (*F*_1,24_ = 20.21; *P* = 0.0001; panel a).

We report results of FRB based on two estimation approaches (i.e., based on excavated soil monoliths and on sequential coring), and of FRP and turnover rate based on sequential coring method. Fine root biomass (≤ 2 mm diameter) in the control plots averaged 169 ± 16 (± standard error (SE) of the mean) g m^−2^ in the top 10 cm soil depth as determined from the excavated soil monoliths (Supplementary Fig. 1). In the first year of nutrient addition (2019), FRB was significantly lower by 36% in the N addition treatment compared with non-N addition treatment (*F*_1,24_ = 20.21; *P* = 0.0001; Fig. 2a; Supplementary Table 1 and Supplementary Fig. 1), which remained almost unchanged (35%) after the second year (2020) of nutrient addition (no year effect: *F*_1,24_ = 1.09; *P* = 0.306; Supplementary Table 1 and Supplementary Fig. 1). No significant FRB response to P or K additions were observed from the excavated soil monoliths (Fig. 2b,c). In the top 100 cm soil depth, about 60% of the FRB occurred in the top 0–10 cm soil depth, whereas 80% occurred in the top 30 cm of the soil profile (Supplementary Fig. 2). We found no relationship between root biomass and tree density or size of neighbouring trees (either in 1 m or 2 m radius) to the measurement locations.

**Figure 3:**
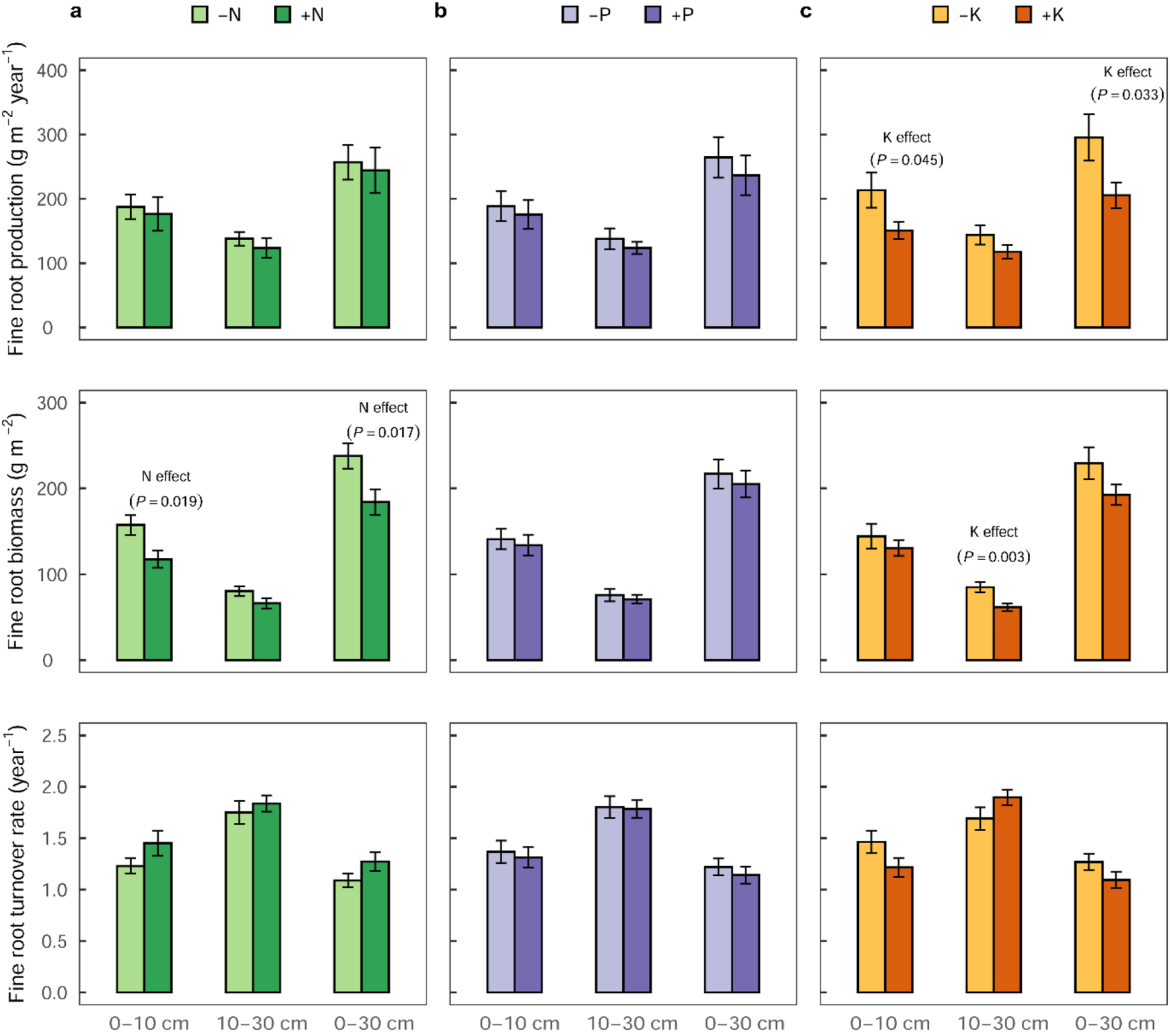
Nutrient addition effect on fine root production, biomass, and turnover rate (mean ± SE, n = 16 plots), measured by sequential coring method (at 0 − 10 cm and 10 − 30 cm soil depths) in the second year of the full factorial N-P-K experiment in the Budongo Forest Reserve, Uganda. Fine root production responses in (**a**) − N (Control, P, K, PK) compared to + N (N, NP, NK, NPK) addition plots, (**b**) − P (Control, N, K, NK) compared to + P (P, NP, PK, NPK) addition plot, and (**c**) − K (Control, N, P, NP) compared to + K (K, NK, PK, NPK) addition plots. In (**a**), N additions decreased fine root biomass in the top 0−10 cm (*F*_1,24_ = 6.31;* P* = 0.019) and in the combined 0−30 cm (*F*_1,24_ = 6.63;* P* = 0.017) soil depth, with no significant effect on fine root production, and turnover rate. In (**b**), no effect of P additions on fine root production, biomass, and turnover rate was found. In (**c**), K additions decreased fine root production in the top 0−10 cm (*F*_1,24_ = 4.47;* P* = 0.045) and in the 0−30 cm (*F*_1,24_ = 5.14;* P* = 0.033) soil depths, and decreased fine root biomass in the 10−30 cm (*F*_1,24_ = 10.75;* P* = 0.003) soil depth.

Additionally, FRB determined from the sequential coring technique (SC) decreased by 25% with N additions in the top 0–10 cm (*F*_1,24_ = 6.31;* P* = 0. 019) and by 23% in the combined 0–30 cm (*F*_1,24_ = 6.63;* P* = 0. 017) soil depths (Fig. 3a and Supplementary Table 2), corroborating the results from the excavated soil monolith approach (Fig. 2a). A significant decrease (by 27%) in FRB in response to K additions was observed only at the 10–30 cm soil depth (*F*_1,24_ = 10.75;* P* = 0. 003; Fig. 3c and Supplementary Table 2). No significant response to P additions were observed from the SC-based FRB (Fig. 3b and Supplementary Table 2). The SC-based FRB averaged 147 ± 20 g m^−2^ in the 0‒10 cm soil depth, 95 ± 14 g m^−2^ in the 10‒30 cm, and 242 ± 31 g m^−2^ in the combined 0‒30 cm soil depth (Supplementary Fig. 3).

Fine root production (FRP) decreased with depth and averaged 170 ± 17 g m^−2^ year^−1^ in the top 0‒10 cm soil depth, 140 ± 24 g m^−2^ year^−1^ in the 10‒30 cm depth, and 234 ± 24 g m^−2^ year^−1^ in the combined 0‒30 cm depth of the control plots (Supplementary Fig. 3). In the top 30 cm soil depth, FRP (1.2 ± 0.1 Mg C ha^−1^ year^−1^) represented 13% of annual NPP (9.5 ± 0.9 Mg C ha^−1^ year^−1^) at this site (Table 1). The addition of K was associated with decreased annual fine root production by 29% in the top 0–10 cm soil depth (*F*_1,24_ = 4.47;* P* = 0. 045; Fig. 3c), and 30% in the 0–30 cm soil depth (*F*_1,24_ = 5.14;* P* = 0. 033; Fig. 3c), with no significant treatment effect observed in the 10–30 cm soil depth (Supplementary Table 2 and Supplementary Fig. 2). Nitrogen and P additions showed no significant effect on FRP (Fig. 3, Supplementary Table 2, and Supplementary Fig. 3).

Fine root turnover rates (FRT) were not significantly affected by nutrient additions (Supplementary Table 2) but tended to increase with soil depth, and higher in N addition than non-N addition treatment (Fig. 3). Turnover rates averaged 1.3 ± 0.2 year^−1^ for the top 0–10 cm soil depth, 1.5 ± 0.2 year^−1^ for 10–30 cm soil depth and 1.0 ± 0.1 year^−1^ for a combined 0–30 cm soil depth in the control plots (Supplementary Fig. 3). There were no treatment interaction effects found in all parameters (i.e., FRB, FRP, and FRT) analyzed (Supplementary Tables 1 and 2).

## Discussion

Fine root production (1.2 ± 0.1 Mg C ha^−1^ year^−1^) was within range of values reported from other tropical forests (Table 1) and identical to FRP values reported from Central Amazonian lowland forests^8^. Fine root biomass (1.2 ± 0.2 Mg C ha^−1^) was relatively lower than those reported from tropical forests in Ghana^58^, Panama^42,50^, and French Guiana^57^, possibly reflecting site differences in stand age^59^, root distribution, and soil fertility^60^. The identical FRP and FRB at our site (FRT = 1.0 ± 0.1 year^−1^, thus, fine root lifespan of about 365 days) implies a replacement of the entire fine root network on an annual basis suggesting a steady state ecosystem, where FRP and fine root mortality occur in equal proportions^61^ and root growth is likely adapted to the site’s nutrient supply. Moreover, the extent to which nutrient availability constrain different components of NPP (above-, and below-ground) and which single or multiple nutrients are responsible for these limitations remain largely unresolved^38,57^, partly due to the varied responses of NPP or its components to nutrient additions in different tropical forests (Table 1). As an example, several methods of estimating FRP are discussed in literature^40^, however, the lack of consensus for a single established approach for the tropics^40^ and site-specific soil fertility^57,62^ may contribute to the contrasting responses of FRP or FRB to nutrient additions^50^ in different forests. In our experiment, the response of FRB to nutrient additions was consistent between the two estimation methods (i.e., monolith-based FRB and SC-based FRB; Figs. 2a and 3a respectively). Furthermore, there are also limited data on root productivity responses to nutrient additions and thus hampering consideration in vegetation models^62^, and direct comparisons within Afrotropical forests only. In the following, we discuss the first direct responses of fine root growth to nutrient additions in a rather understudied natural tropical system.

### Nitrogen addition effects on fine roots

The response of stand-level FRB to N additions was rather rapid^10,42,63^ and was evident in the first year of our experiment (Fig. 2a). The rapid reductions in FRB associated with N addition provide direct evidence of an alleviated N limitation in this ecosystem and highlights the high plasticity of fine root response to elevated N availability in the short-term. Building new roots and maintaining metabolically older roots are highly costly processes and require large resource (i.e., energy and nutrients) investments by the plants^42^. Our results demonstrate that, trees in this ecosystem scaled-back their large fine root biomass allocations, in the first year, as N became adequately available^43,45,47,49^. This explains why we did not detect any significant response in FRP to N additions (Fig. 3a) nor measure significant further declines in FRB in the second year (Supplementary Table 1 and Supplementary Fig. 1). Although not significant, the tendency of increased turnover rate in N addition treatment support the idea that FRT rate should be higher in fertile than infertile soils due to the higher root respiration cost associated with increased nutrient availability^42^. These responses of fine root growth (expressed in FRB, FRT, and FRP) to N additions are consistent with the increased stem growth associated with N additions in the second year of the experiment^9^, which may represent a reallocation of root C for stem growth. Soil total N in the top 30 cm depth of our site (2.0 g N kg^−1^) was similar to that of the Panamanian (Gigante) old-growth tropical lowland forest (2.2 g N kg^−1^)^32^, where N and K additions together enhanced stem growth of saplings and marginally decreased stand-level FRB^10^, thereby highlighting their potential limitation. Conversely, no significant FRB response to N additions were observed in the first two years of nutrient additions in a Costa Rican tropical lowland forest^64^, likely because this forest’s soils had a relatively higher indicator of N bioavailability than our site, hence the lack of response to N additions^64^. Moreover, our results support similar reductions in root productivity and belowground C allocation following an alleviated N limitation in temperate and boreal forests^49^ respectively. In our experiment, given the absence of a relationship between FRB and the density or sizes of the neighbouring trees, the spatial distribution of FRB was most likely driven primarily by soil nutrient (such as N) availability at our site. Considering that N limitation is clearly evident at our site, the proposition that N availability is in excess of plant demand in tropical forests is hereby not supported.

### Potassium addition effects on fine roots

The reduction in FRB and FRP in response to K additions in this experiment suggest an alleviated K limitation and is in agreement with observations from other tropical nutrient addition experiments, where FRB or FRP likewise decreased in response to K additions^10,42,50,65^. Whilst the lack of FRT response to added K may be explained by the paralleled decrease in FRB and FRP, in which case, less FRP account for less FRB on fertile soils^42^, our results contrasts observations from a largescale nutrient addition experiment in Panama where K addition decreased FRB and increased FRT^42^. Nevertheless, our results support the hypothesis that K likely limit ecosystem-level plant function on highly weathered soils. Similar to N addition effect, the reduction in FRP and FRB under K additions was consistent with a 46% stem growth increase associated with K additions among semi-deciduous trees^9^ and K limitation of leaf litter production in our experiment^16^, further substantiating that these nutrients indeed limit both above- and belowground processes in this ecosystem. Moreover, K was the most resorbed nutrient at our experimental site^16^, suggesting that trees generally adopted a conservative strategy towards K-use due to its limited availability. This limitation may have resulted from leaching loses of K, facilitated by our site’s sandy soil texture (Table 2), as well as the high mobility and dissolvability of K^+^ ions^36^. Although the role of K have been largely overlooked in natural ecosystem processes, increasing reports^9,10,42,66–69^ and our findings suggest that limitations by K and other base cations^29^ on ecosystem productivity and function could be far more widespread in most tropical forests than suspected. Such K limitations are yet uncaptured in current models that predict C-sink potential of tropical forests and consequently may be overestimating the capacity of these sinks. Potassium is particularly recognized for its role in mitigating the effect of drought on plant function^16^ by enhancing water-use efficiency through effective regulation of leaf stomatal conductance^24,70–72^. In view of this, the greater possibility of K limitation in seasonally dry tropical forest and its mechanistic role in the resilience of these forests deserve further attention.

### Phosphorus addition effects on fine roots

As hypothesized, no response in FRB or FRP with P addition was observed in the short-term (2019‒2020; Figs. 2b and 3b). This result agrees with observations in a Costa Rican N–P addition experiment where no root growth responses to P additions were observed in the short-term (< 3 years)^64^. In contrast, P additions increased root productivity and/or root diameter^8,51^ in central Amazonian forests growing on relatively P-impoverished soils^52–54^ and in Costa Rican (Guanacaste) tropical dry secondary forest^73^. Considering the relatively low P-levels of these forest sites^8,27^, increased root productivity can be expected when prevailing P limitation is not alleviated by experimental P inputs. The lack of fine root growth response to P additions in this experiment was not particularly surprising given the near-neutral soil pH at the site (Table 2). Under these conditions, P is not fixed by hydroxides of Fe and Al, and hence sufficient P should be available for plant uptake^74^. Indeed, soil total P at our site (557 mg P kg^−1^ in the top 30 cm depth; Table 2) was higher than most part of central and eastern Amazonia where plant growths were responsive to P additions^8^. Therefore, P limitation of fine root growth at this site was indeed unlikely nor is the widely hypothesized P limitation of primary productivity on strongly weathered tropical soils^8,14,25^ supported.

## Conclusion

This two-year nutrient addition experiment provided key insights into how and which nutrients control belowground productivity in this ecosystem. Our results revealed that, N and K but not P availability controlled fine root growth in this natural ecosystem, in support of the multiple nutrient limitation concept and thus challenging the Liebig’s law of the minimum (the traditional view that ecosystems are limited by a single nutrient at any given time). The addition of N reduced FRB by up to 36% in the first year of the experiment which did not change after the second year, suggestive of an alleviated ecosystem-scale N limitation. Similarly, limitations by K are clearly indicated by the consistent reductions in FRB (by 27%) and FRP (by 30%) associated with K additions. These rapid reductions in fine root growth suggests a scaled-back C allocation to fine roots as limiting nutrients become adequately available. Whereas the impact of sustained nutrient additions on fine roots remains to be evaluated, it can be expected, that the N and K limitation of productivity in this ecosystem would favour N-fixing plant species as well as plant species with enhanced K mobilisation strategies, this can potentially cause shifts in species composition and distribution in the long-term. Moreover, given that Africa is yet largely unindustrialized (i.e., lesser nutrient depositions), the unexpectedly fast response of this ecosystem to nutrient additions suggests high plasticity or rather high responsiveness of Afrotropical forests to heightened nutrient depositions expected in the future. Our data suggest that these limitation by N and K can have a stronger implication on CO_2_ assimilation and on ecosystem resilience in these forests yet, to the best of our knowledge, K limitation of productivity remains uncaptured in current biogeochemical models. Indeed, the rarity of field based nutrient manipulation experiments, particularly in Afrotropical forests, would continue to pose a major challenge in the identification and representation of limiting nutrients in constraining current models. More of these experiments are required in the African tropical forest region to capture the broad range of factors that directly or indirectly control the responses of primary productivity to elevated nutrient inputs and future environmental perturbations.

## Materials and methods

### Study site description

We conducted this experiment in the Budongo Forest Reserve in northwestern Uganda (1° 44′ 28.4′′ N, 31° 32′ 11.0′′ E; mean elevation: ~ 1050 m above sea level). Permissions to conduct the experiment were granted by the Ugandan National Council for Science and Technology (UNCST; NS 619) and the Ugandan Wildlife Authority (COD/96/02). All methods were carried out in line with the relevant guidelines. No voucher specimens were compiled during the field campaigns.

This humid, semi-deciduous tropical forest (Fig. 1a) at Budongo is situated on an uplifted shield, specifically, on a Precambrian gneissic-basaltic basement complex^75^. Mean annual air temperature and precipitation was 22.8 ± 0.1 °C and 1670 ± 50 mm respectively (2000–2019; Budongo Conservation Field Station), with nutrient depositions from rainfall measuring 8.5 kg N ha^–1^ year^–1^, 0.03 kg P ha^–1^ year^–1^ and 4.3 kg K ha^–1^ year^–1^^9^. Soils at the site are well-drained sandy loam (> 50% sand; Fig. 1b and Table 2), highly weathered and are classified as Lixisols^39^, characterized by high soil base saturation, calcium-dominated cation exchange capacity, and a near-neutral soil pH (Table 2). Although the high soil calcium content contrast other sites in the Congo basin enclave^11^, it is likely derived from the weathering of geological parent material^9^ as well as ash deposition from either regional biomass burning or historic volcanic activity^76–78^.

Vegetation at the experimental site is species-rich and diverse (126 tree species; Shannon-diversity index H’: 2.53 ± 0.04). Among trees ≥ 10 cm dbh, 6% represented nitrogen-fixing trees in stem abundance, which accounted for 16% of the forest’s basal area^9^. Leaf litterfall at this site averaged 8.5 ± 0.3 Mg ha^−1^ year^−1^, leaf area index averaged 3.3 ± 0.0 m^2^ m^−2^ (determined in April 2018 and November 2019), and wood density averaged 0.58 g cm^−3^ in the control plots, with tree heights reaching up to 50 m. Annual rates of nutrient input through leaf litterfall were 212 ± 5 kg N ha^−1^ year^−1^, 11 ± 0 kg P ha^−1^ year^−1^, 77 ± 2 kg K ha^−1^ year^−1^, 278 ± 15 kg Ca ha^−1^ year^−1^, and 29 ± 2 kg Mg ha^−1^ year^−1^. The six most dominant species of all trees ≥ 10 cm DBH contributing 63% of stem abundance at the experimental site include: *Funtumia elastica *(24%),* Celtis mildbraedii *(15%),* Cynometra alexandri *(6%),* Celtis durandii *(6%),* Celtis zenkeri *(6%), and* Lasiodiscus mildbraedii *(6%). Despite a selective logging history (1952–1954) for economic species, the site has remained undisturbed for nearly 60 years now^79^. The most noticeable effect of this past logging was an increased species richness compared to an unlogged compartment^79^ and a higher abundance of mid-stage succession tree species (e.g. *Funtumia elastica*). Typical of natural ecosystems, tree density decreased with increasing dbh classes: 5938 ± 269 for 1–5 cm, 627 ± 30 for 5–10 cm, 514 ± 13 for 10–30 cm and 108 ± 5 for > 30 cm.

### Experimental design

In 2018, we established a 2^3^ full factorial NPK experiment with eight treatments (N, P, K, NP, NK, PK, NPK and control; Fig. 1c). These treatments had four replicates each and were randomly assigned to a total of 32 plots (40 × 40 m each), which are at least 40 m apart. Within each 40 × 40 m plot, we also laid out a 30 × 30 m (effective plot size) and sixteen 10 × 10 m quadrats to facilitate fertilizer addition. For comparability, our experimental design followed a similar study in Panama^10^. Nitrogen was added as urea ((NH_2_)_2_CO; 125 kg N ha^−1^ year^−1^), P as triple superphosphate [Ca(H_2_PO4)_2_; 50 kg P ha^−1^ year^−1^] and K as muriate of potash [KCl; 50 kg K ha^−1^ year^−1^] in each 40 × 40 m plot area. Pre-packaged fertilizers for each 10 m × 10 m quadrat were mixed with soil adjacent the plots as filler materials and broadcasted by hand, walking forward and back and subsequently changing directions (north to south and east to west). We fertilized four times (beginning from 17th May 2018) each year in equal doses during the wet season. All response measurements were conducted in the central 30 m × 30 m (900 m^2^) of each plot^10,51^ to reduce edge effects.

### Fine-root biomass and productivity measurements

We quantified fine-root biomass in the top 0–10 cm soil depth by excavating soil monoliths (20 × 20 × 10 cm depth) at six randomly selected grid locations in each of the 32 sampling plots (i.e., 192 samples in each sampling year) at the end of the first (June 2019) and second (June 2020) year of the experiment (Fig. 2). Fine roots (≤ 2 mm diameter) from each soil monolith were hand-washed, and oven-dried until constant mass at 60 °C at least 24 h. We also analysed the spatial variability of root biomass against possible controlling factors such as distance to the nearest tree (≥ 10 cm DBH) and tree density^9^.

Fine root production was estimated using sequential coring technique in the second year of the experiment; although laborious, this technique is known to give the most reliable results^40^. We used a sharp steel root corer (diameter = ~ 35 mm) to sample at two random grid locations per plot in the top 30 cm soil depth (separately at 0–10 cm and 10–30 cm), where about 80% of roots were found at this site (Supplementary Fig. 2, and thus representative of the total fine root biomass in this forest. Root biomass samples were taken every three months (May 2019 (initial measurement), August 2019 (3 months), November 2019 (6 months), February 2020 (9 months), and May 2020 (12 months)), hand-washed over a 2-mm sieve, then oven-dried and dried-mass determined. For each sampling depth, we calculated fine root production using the ‘minimum–maximum’ method^80^, by taking the difference between the maximum and minimum fine root biomass during an entire year’s measurement period. To determine fine root production for the entire 0–30 cm, we summed the root biomass within the 0–30 cm soil depth (i.e., 0–10 cm and 10–30 cm) on each sampling period, then applied the maximum-minimum approach to estimate fine root production across the sampling periods (Fig. 3). Fine root turnover rate (year^−1^; the inverse of turnover time) was calculated by dividing the annual fine root production by the mean fine root biomass^61^ (averaged across the five measurement periods).

### Soil physicochemical characteristics

Soil biochemical characteristics were measured in April 2018 prior to initial nutrient addition. Soil samples were taken from 10 random locations per plot at 0–10 cm and 10–30 cm soil depth in all 32 plots. Soil organic carbon (SOC) and total N were analyzed using a CN elemental analyzer (VARIO EL Cube, Elementar Analysis Systems GmbH, Hanau, Germany). Exchangeable cations were determined by percolating the soil samples with unbuffered 1 M NH_4_Cl and cation concentrations in percolate were analyzed using the inductively coupled plasma-atomic emission spectrometer (ICP-AES; iCAP 6300 Duo VIEW ICP Spectrometer, Thermo Fischer Scientific GmbH, Driesch, Germany). Soil pH was analyzed in 1:2.5 of soil-to-distilled water ratio. Soil texture for each plot was determined from a composite sample using the pipette method after iron oxide and organic matter removal^81^. Soil bulk density (corrected for stone content) was measured from soil pits dug next to each plot using the core method^82^.

### Statistical analyses

Soil physical and biochemical characteristics did not significantly differ among plots proir to nutrient addition^9^. We used linear mixed-effect (LME) models to test the effect of nutrient addition treatments and their interaction in the full factorial N × P × K experimental design on the repeated fine root biomass measurements (‘nlme’ package). For the LME model, the absence/presence of each of the nutrients (N, P, and K) and measurement year (1st year, 2nd year) were the fixed effects^10^, and replicate plots as random effect. The significance of the fixed effect was evaluated using analysis of variance (ANOVA)^83^. Residual plots met the assumptions of normality and homogeneity of variance; therefore, no data transformation was necessary. To compare the responses of fine root biomass obtained by the two sampling methods (excavated soil monolith and sequential coring) and to obtain fine root biomass response at soil depth deeper than 0–10 cm, we also analysed the mean fine root biomass (averaged over the five measurement periods), measured from the sequential cores. Fine root production, turnover rate and mean fine root biomass (determined from the sequential coring) were tested for treatment effects using factorial ANOVA (lm function). All parameters were first tested for normal distribution (Shapiro–Wilk’s test) and equality of variance (Levene’s test). In all tests, statistical significance was set at *P* ≤ 0.05. All statistical analyses were performed using the statistical package R version 4.3.2^84^.

### Supplementary Information

Supplementary Information.

## Data Availability

Data are available from Göttingen Research Online/Data at 10.25625/XFBHCS (ref.^87^).
