# Supplementary material for: Nitrogen and potassium limit fine root growth in a humid Afrotropical forest
Source: Sci Rep. 2024 Jun 7;14:13154. doi: 10.1038/s41598-024-63684-7 (PMC11161472; doi:10.1038/s41598-024-63684-7)
Supplement: Supplementary file 1 — Supplementary Information. [file 41598_2024_63684_MOESM1_ESM.docx]

**SUPPLEMENTARY INFORMATION**

Raphael Manu, Edzo Veldkamp David Eryenyu, Marife D. Corre, and Oliver van Straaten Nitrogen and potassium limit fine root growth in a humid Afrotropical forest

**Supplementary Table 1**. Analysis of variance on the linear mixed-effect models for testing the effects of nutrient addition and measurement year on fine root biomass, measured in June 2019 and June 2020 using soil monoliths (20 cm × 20 cm × 10 cm depth), in full factorial N-P-K experiment in the Budongo Forest Reserve, Uganda.

| Treatment | numDF | denDF | *F*-statistics | *P*-value |
| --- | --- | --- | --- | --- |
| N | 1 | 24 | **20.206** | **0.0001** |
| P | 1 | 24 | 0.068 | 0.796 |
| K | 1 | 24 | 0.163 | 0.690 |
| Year | 1 | 24 | 1.095 | 0.306 |
| N × P | 1 | 24 | 1.313 | 0.263 |
| N × K | 1 | 24 | 0.488 | 0.491 |
| P × K | 1 | 24 | 0.001 | 0.979 |
| N × P × K | 1 | 24 | 0.244 | 0.626 |
| N × year | 1 | 24 | 0.003 | 0.960 |
| P × year | 1 | 24 | 0.231 | 0.635 |
| K × year | 1 | 24 | 0.615 | 0.441 |
| N × P × year | 1 | 24 | 0.538 | 0.470 |
| N × K × year | 1 | 24 | 0.029 | 0.867 |
| P × K × year | 1 | 24 | 1.866 | 0.185 |
| N × P × K × year | 1 | 24 | 1.918 | 0.179 |

**Supplementary Table 2.** Analyses of variance of fine root production, fine root biomass and fine root turnover rate, measured by sequential coring (in May 2019, August 2019, November 2019, February 2020, and May 2020) at 0‒10 cm, 10‒30 cm and entire 0‒30 cm soil depths, in full factorial N-P-K experiment in the Budongo Forest Reserve, Uganda.

| Treatment | numDF | denDF | Fine root production | | Fine root biomass  (based on sequential coring) | | Fine root turnover rate | |
| --- | --- | --- | --- | --- | --- | --- | --- | --- |
|  |  |  | *F*-statistics | *P*-value | *F*-statistics | *P*-value | *F*-statistics | *P*-value |
| *0−10 cm soil depth* | | | | | | | | |
| N | 1 | 24 | 0.143 | 0.709 | **6.309** | **0.019** | 2.500 | 0.127 |
| P | 1 | 24 | 0.185 | 0.671 | 0.199 | 0.660 | 0.154 | 0.699 |
| K | 1 | 24 | **4.465** | **0.045** | 0.752 | 0.394 | 3.143 | 0.089 |
| N × P | 1 | 24 | 4.118 | 0.054 | 0.311 | 0.582 | 3.334 | 0.080 |
| N × K | 1 | 24 | 0.549 | 0.466 | 0.139 | 0.713 | 0.392 | 0.537 |
| P × K | 1 | 24 | 0.231 | 0.635 | 0.001 | 0.976 | 0.226 | 0.639 |
| N × P × K | 1 | 24 | 2.075 | 0.163 | 2.880 | 0.103 | 0.115 | 0.738 |
| *10−30 cm soil depth* | | | | | | | | |
| N | 1 | 24 | 0.744 | 0.397 | 3.982 | 0.057 | 0.427 | 0.520 |
| P | 1 | 24 | 0.716 | 0.406 | 0.483 | 0.494 | 0.018 | 0.895 |
| K | 1 | 24 | 2.513 | 0.126 | **10.751** | **0.003** | 2.391 | 0.135 |
| N × P | 1 | 24 | 3.925 | 0.059 | 1.681 | 0.207 | 1.787 | 0.194 |
| N × K | 1 | 24 | 2.953 | 0.099 | 0.781 | 0.386 | 1.062 | 0.313 |
| P × K | 1 | 24 | 3.957 | 0.058 | 3.197 | 0.086 | 0.746 | 0.396 |
| N × P × K | 1 | 24 | 0.282 | 0.600 | 0.274 | 0.605 | 1.394 | 0.249 |
| *0−30 cm soil depth* | | | | | | | | |
| N | 1 | 24 | 0.103 | 0.751 | **6.628** | **0.017** | 2.915 | 0.101 |
| P | 1 | 24 | 0.496 | 0.488 | 0.328 | 0.572 | 0.531 | 0.473 |
| K | 1 | 24 | **5.137** | **0.033** | 3.114 | 0.090 | 2.671 | 0.115 |
| N × P | 1 | 24 | 3.742 | 0.065 | 0.740 | 0.398 | 3.651 | 0.068 |
| N × K | 1 | 24 | 1.763 | 0.197 | 0.337 | 0.567 | 1.699 | 0.205 |
| P × K | 1 | 24 | 0.341 | 0.565 | 0.339 | 0.566 | 0.004 | 0.950 |
| N × P × K | 1 | 24 | 2.166 | 0.154 | 1.225 | 0.279 | 0.534 | 0.472 |


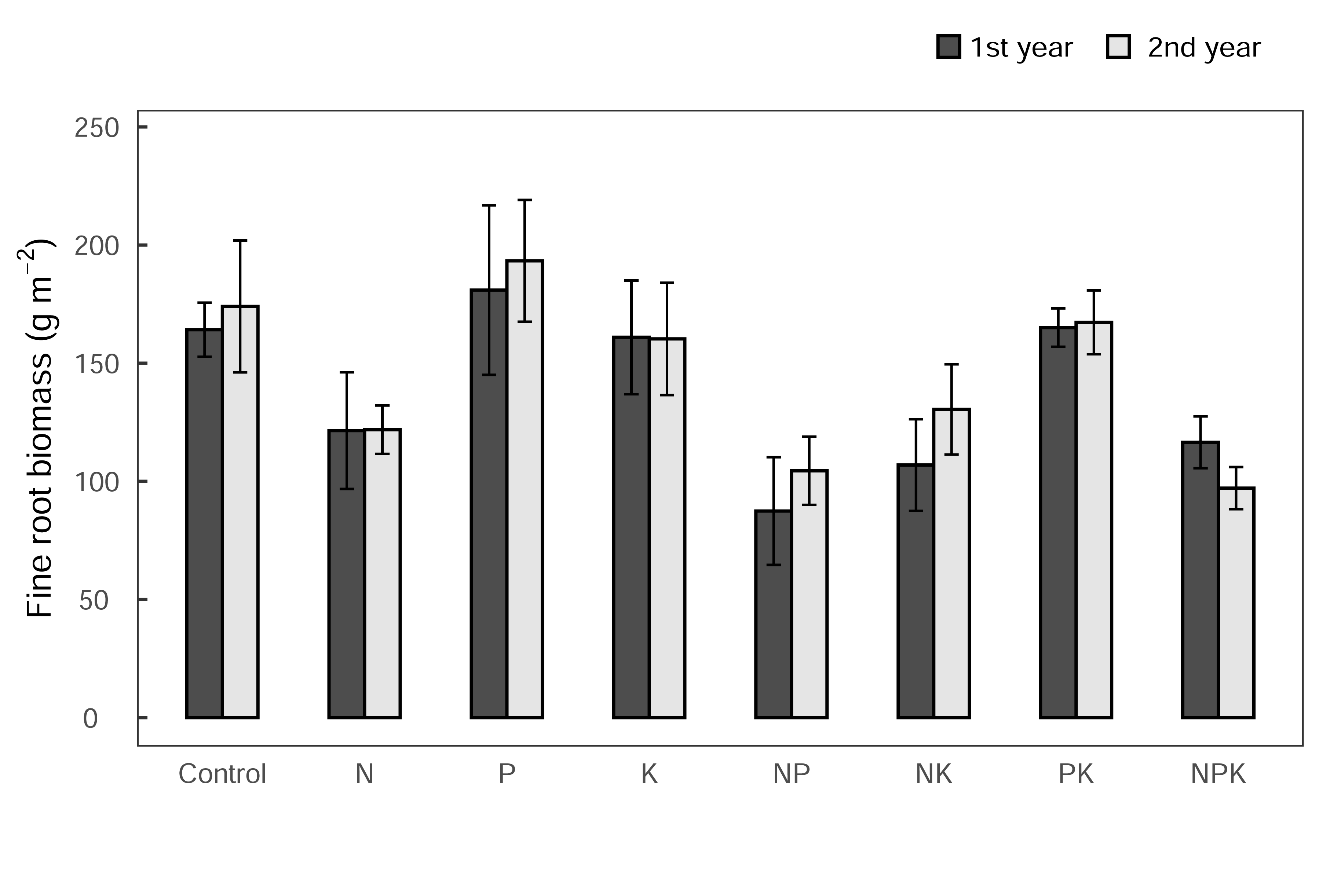


**Supplementary Fig. 1.** Nutrient addition effect on fine root biomass (mean ± SE, n = 4 plots), measured from six randomly located soil monoliths (20 cm × 20 cm × 10 cm) per plot after the first year (June 2019) and second year (June 2020) of the full factorial N-P-K experiment in the Budongo Forest Reserve, Uganda.

**Supplementary Fig. 2**. Fine root biomass (mean ± SE; n = 4 plots) at different soil depths, measured in the control plots of the full factorial N-P-K experiment in the Budongo Forest Reserve, Uganda.


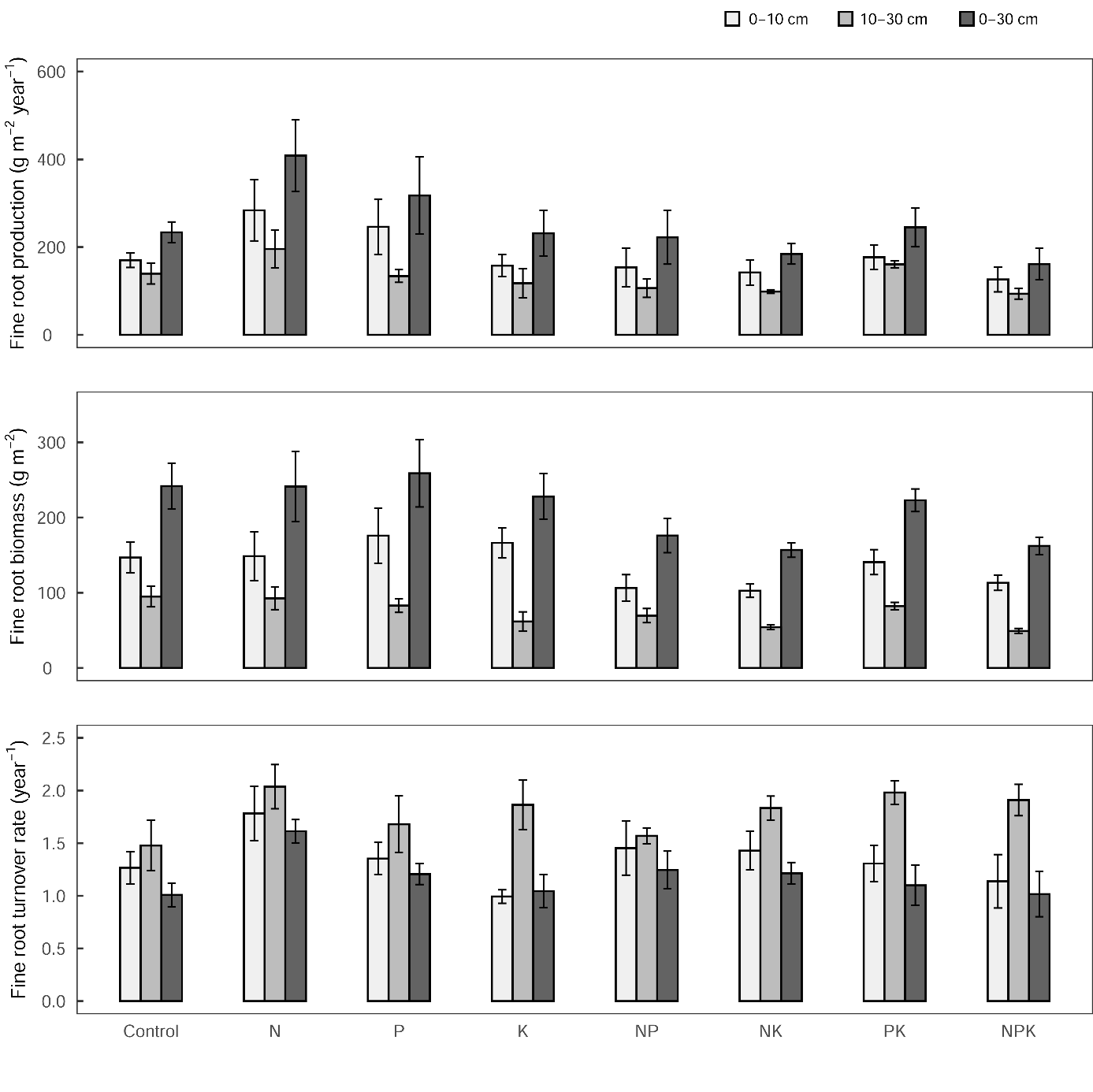
**Supplementary Fig. 3**. Nutrient addition effect on fine root production, biomass, and turnover rate (mean ± SE, n = 4 plots), measured by sequential coring method (at 0−10 cm and 10−30 cm soil depths) in the second year (May 2019, August 2019, November 2019, February 2020, and May 2020) of the full factorial N-P-K experiment in the Budongo Forest Reserve, Uganda.
